# Semipurified Ethyl Acetate Partition of Methanolic Extract of* Melastoma malabathricum* Leaves Exerts Gastroprotective Activity Partly via Its Antioxidant-Antisecretory-Anti-Inflammatory Action and Synergistic Action of Several Flavonoid-Based Compounds

**DOI:** 10.1155/2017/6542631

**Published:** 2017-01-11

**Authors:** Noor Wahida Ismail Suhaimy, Ahmad Khusairi Noor Azmi, Norhafizah Mohtarrudin, Maizatul Hasyima Omar, Siti Farah Md. Tohid, Manraj Singh Cheema, Lay Kek Teh, Mohd. Zaki Salleh, Zainul Amiruddin Zakaria

**Affiliations:** ^1^Department of Biomedical Science, Faculty of Medicine and Health Science, Universiti Putra Malaysia, 43400 Serdang, Selangor, Malaysia; ^2^Department of Pathology, Faculty of Medicine and Health Science, Universiti Putra Malaysia, 43400 Serdang, Selangor, Malaysia; ^3^Phytochemistry Unit, Herbal Medicine Research Centre, Institute for Medical Research, Jalan Pahang, 50588 Kuala Lumpur, Malaysia; ^4^Integrative Pharmacogenomics Institute (iPROMISE), Universiti Teknologi MARA, Level 7, FF3 Building, 42300 Puncak Alam, Selangor, Malaysia

## Abstract

Recent study has demonstrated the gastroprotective activity of crude methanolic extract of* M. malabathricum *leaves. The present study evaluated the gastroprotective potential of semipurified extracts (partitions): petroleum ether, ethyl acetate (EAMM), and aqueous obtained from the methanolic extract followed by the elucidation of the gastroprotective mechanisms of the most effective partition. Using the ethanol-induced gastric ulcer assay, all partitions exerted significant gastroprotection, with EAMM being the most effective partition. EAMM significantly (i) reduced the volume and acidity (free and total) while increasing the pH of gastric juice and enhanced the gastric wall mucus secretion when assessed using the pylorus ligation assay, (ii) increased the enzymatic and nonenzymatic antioxidant activity of the stomach tissue, (iii) lost its gastroprotective activity following pretreatment with N-omega-nitro-L-arginine methyl ester (L-NAME; NO blocker) or carbenoxolone (CBXN; NP-SH blocker), (iv) exerted antioxidant activity against various in vitro oxidation assays, and (v) showed moderate in vitro anti-inflammatory activity via the LOX-modulated pathway. In conclusion, EAMM exerts a remarkable NO/NP-SH-dependent gastroprotective effect that is attributed to its antisecretory and antioxidant activities, ability to stimulate the gastric mucus production and endogenous antioxidant system, and synergistic action of several gastroprotective-induced flavonoids.

## 1. Introduction

Gastric ulcer is an injury that occurs in the stomach lining. The ulcer disrupts the mucosa integrity of the stomach with extension beyond the submucosa into the muscularis mucosa due to the active inflammation. 5% of human population suffers from gastric ulcer [1]. Current treatments available to treat ulcer work by reducing acid secretions or increasing mucosal protection such as proton pump inhibitor (PPI), histamine antagonist (H2) blocker, and antacids. Nevertheless, it comes with undesirable side effects such as gynecomastia, arrhythmias, and haematopoietic changes [2].

Hence, there is a shift in interest in using natural products as an alternative source of medicine with promising results in treating diseases. With regard to gastric ulcer treatments, various studies have shown the ability of plant-based extracts to exert gastroprotective activity such as* Bauhinia purpurea* [3],* Muntingia calabura *[4], and* Annona reticulate *[5].

*Melastoma malabathricum* L. belongs to the family Melastomataceae. This shrub is commonly found throughout Southeast Asia region including Malaysia and is known among the Malay community as “Senduduk.” Various parts of* M. malabathricum* have been used to treat different types of diseases with the leaves, in particular, have been used to treat gastric ulcers [6]. Various other parts of* M. malabathricum* in various forms of extraction have been reported to exhibit various types of pharmacological activities such as antibacterial, antiviral, antinociceptive, anti-inflammatory, antipyretic, antioxidant, anticoagulant, inhibitor of platelet-activating factor, antidiarrheal, and wound healing activities [6]. We have recently reported on the antiulcer potential of the methanolic extract of* Melastoma malabathricum* (MEMM) leaves [7]. In an attempt to identify the bioactive compound(s) that is responsible for MEMM-exerted antiulcer activity, the present study was designed to use the semipurified extracts, namely, petroleum ether (PEMM), ethyl acetate (EAMM), and aqueous (AQMM) partitions obtained through the partitioning of water-dissolved MEMM using petroleum ether followed by ethyl acetate to determine the gastroprotective activity of these partitions and to elucidate the mechanisms of gastroprotection exerted by the most effective partition and thereafter to identify the compound(s) in the most effective partition.

## 2. Materials and Methods

### 2.1. Plant Materials

*M. malabathricum* leaves were collected from their natural habitat in Serdang, Selangor, Malaysia, between August and September 2013 and identified by a botanist (Dr. Shamsul Khamis) from the Institute of Bioscience (IBS), Universiti Putra Malaysia (UPM), Serdang, Selangor, Malaysia. A voucher specimen (SK 1095/05) was issued and deposited in the Herbarium of the Laboratory of Natural Products, IBS, UPM.

### 2.2. Preparation of Methanol Extract of* M. malabathricum* and the Various Semipurified Extracts

Eight hundred grams of dried* M. malabathricum* leaves was grinded and soaked in methanol for 72 h at room temperature and this was repeated three times. The methanol supernatant was collected, pooled together, and then evaporated to yield approximately 40 g of dried crude MEMM [7]. The dried crude extract was first added with distilled water (ratio of 1 : 20; m/v) and then shaken to dissolve them well and then successively partitioned with the same volume of petroleum ether followed by ethyl acetate as described elsewhere [8]. The process of partitioning was repeated for the respective solvent until no changes in color could be seen in the supernatant. Each supernatant was then pooled together and evaporated leading to the yield of semipurified extracts of petroleum ether, ethyl acetate, and distilled water (aqueous partition).

### 2.3. Phytochemical Screening of Various Semipurified Extracts of MEMM

Each partition was subjected to phytochemical screening according to standard conventional screening tests as described by Ikhiri et al. [9]. The phytochemical screening was performed to identify the presences of alkaloids, flavonoids, triterpenes, tannins, saponins, and steroids by using 100 mg of each partition.

### 2.4. Antioxidant Potential of Various Semipurified Extracts of MEMM Assessed Using Several Oxidation Assays

#### 2.4.1. Superoxide Anion (SOA) Radical Scavenging

The SOA radical scavenging activity was determined according to Liu et al. [10] but with slight modification. A mixture of 3 mL of Tris-HCl buffer (16 mM, pH 8), 1 mL of NBT (50 *μ*M), 1 mL NADH (78 *μ*M), and each of the respective partition (25–50 *μ*g) was first prepared. The reaction was initiated by adding 1 mL of PMS solution (10 *μ*M) and the mixture solution was incubated at 25°C for 5 min. The activity was read at absorbance 560 nm using a spectrophotometer (Shimadzu UV-Vis 1700) against blank samples and using l-ascorbic acid as a control.

#### 2.4.2. 2,2-Diphenyl-1-picrylhydrazyl (DPPH) Radical Scavenging Assay

The DPPH-radical scavenging assay was performed according to the method of Blois [11] but with slight modification. Approximately, 50 *μ*L of each partition (1.0 mg/mL) was loaded and followed by 50 *μ*L of DPPH (1 mM in ethanolic solution) and 150 *μ*L of absolute ethanol was added in 96-well microtiter plate in triplicate. The mixtures were mixed vigorously for 15 s at 500 rpm and incubated at room temperature for 30 min and the absorbance was measured spectrophotometrically at 515 nm.

#### 2.4.3. Oxygen Radical Absorbance Capacity (ORAC) Assay

The ORAC assay was performed according to the method of Huang et al. [12] but with slight modification. The sample was assayed in 96-well plate and was measured every 60 s. The ORAC value was analyzed using MARS Data Analysis Reduction Software.

#### 2.4.4. Total Phenolic Content (TPC)

The TPC of each partition was determined using the Folin-Ciocalteu reagent with gallic acid used as a standard in accordance to the method of Singleton and Rossi Jr. [13] but with slight modification. One milligram of each partition was extracted with 1.0 mL of 80% methanol containing 1.0% hydrochloric acid and 1.0% of distilled water. The mixtures were put on the shaker set at 200 rpm at room temperature and then were centrifuged at 6000 rpm for 15 min. A 200 mL of each supernatant was mixed with 400 mL of the Folin-Ciocalteu reagent (0.1 mL/0.9 mL). The mixtures were incubated at room temperature for 5 min followed by the addition of 400 mL of sodium bicarbonate (60 mg/mL) and incubated at room temperature for 90 min. Absorbance readings were taken spectrophotometrically at 725 nm. The TPC level in each partition was expressed as Gallic acid equivalent (GAE: mg/100 g).

### 2.5. *In Vitro* Effect of Various Semipurified Extracts of* M. malabathricum* against Several Inflammatory Mediators

#### 2.5.1. Xanthine Oxidase (XO) Assay

The XO assay was performed as described by Noro et al. [14]. Ten microlitre of each partition was dissolve in DMSO along with 130 *μ*L potassium phosphate buffer (0.05 M, pH 7.5) and 10 *μ*L of the XO solution and thereafter was incubated for 10 min at 25°C. The assay was measured at absorbance of 295 nm.

#### 2.5.2. Lipoxygenase (LOX) Assay

The LOX activity was determined according to Azhar-Ul-Haq et al. [15]. A mixture of 10 mL of each partition, 160 mL sodium phosphate buffer (0.1 M, pH 8), and 20 mL of soy bean LOX solution was incubated for 10 min at 25°C. The reaction was then initiated by the addition of 10 mL substrate in the form of sodium linoleic acid solution. The absorbance was measured at 234 nm.

### 2.6. Gastroprotective Activity of Various Semipurified Extracts of MEMM Assessed Using Several Gastric Ulcer Models

#### 2.6.1. Experimental Animals

Healthy male Sprague-Dawley rats weighing between 180 and 200 g were used in this study. Rats were maintained under controlled conditions (22 ± 2°C, 12 h light/dark) in the Animal House, Faculty of Medicine, Universiti Putra Malaysia. Free access to food and water was allowed. The experimentation was approved by the Institutional Animal Care and Use Committee, Universiti Putra Malaysia (Ref. UPM/IACUC/AUP-R032/2014).

#### 2.6.2. Ethanol-Induced Gastric Ulcer Model

The gastroprotective activity of each partition was determined against ethanol-induced gastric ulcer in male Sprague-Dawley rats (*n* = 6). The rats were orally administered with 10% DMSO as negative control, 100 mg/kg of ranitidine as positive control or, the semipurified extracts (PEMM, EAMM, or AQMM), in the doses ranging from 50, 250, or 500 mg/kg, for 7 consecutive days prior to the administration of ethanol. Another group of rats received 10% DMSO without ethanol-induced gastric ulcer, which served as the normal control. At the end of the treatment (7th day), gastric ulcers were induced by oral administration of absolute ethanol according to the method described by Zabidi et al. [16]. All rats were euthanized in CO_2_ chamber and the stomachs were removed.

*(1) Macroscopic and Microscopic Evaluations of Treated Stomachs*. The stomachs were dissected along the greater curvature and rinsed with cold saline to remove the contents. The ulcer areas of each stomach were measured and the sum of the area was expressed as the ulcer area (mm^2^) according to Zabidi et al. [16]. The percentage of gastroprotection was calculated using the following equation:

(1) Gastroprotection%=Ulcer  Areacontrol−Ulcer  AreatreatedUlcer  Areacontrol×100.

Each of the stomach samples was fixed with 10% buffered formalin before proceeding with tissue processing. The fixed stomachs were embedded in paraffin block and sectioned using optical rotary microtome to approximately 4 *μ* thickness and stained with hematoxylin and eosin prior to histological evaluation [16].

### 2.7. Pylorus Ligation-Induced Gastric Ulcer Assay on the Most Effective Semipurified Extract of MEMM

The most effective partition determined following the ethanol-induced gastric ulcer assay was further subjected to the pylorus ligation-induced gastric ulcer assay according to the method described by Shay et al. [17] but with some modification. Pylorus ligation was performed 1 h after the administration of the test solutions. The rat's stomach was ligated for 4 h and then the stomach content was collected wherein the volume, pH, free acidity, and total acidity of gastric juice as well as the gastric wall mucus content were measured.

#### 2.7.1. Determination of Gastric Juice's Volume and pH

The gastric content was collected from the stomach of each rat and centrifuged at 2500 rpm for 10 min. The gastric content volume was measured and pH of the supernatant was determined using a pH meter [18].

#### 2.7.2. Determination of Free Acidity and Total Acidity

The free acidity of gastric content was determined by titration of 0.01 N NaOH with methyl orange until the color changes to yellowish. Meanwhile, total acidity was determined by titration with 0.1 N NaOH by using 2% phenolphthalein as acid-base indicator. Total acidity was expressed as mEq/L [18].

#### 2.7.3. Determination of Gastric Wall Mucus

The stomachs were rinsed to clear off any residues and weighed. The stomachs were then immersed in 10 mL of 0.1% Alcian blue in 0.16 M sucrose/0.05 M sodium acetate, pH 5.8 for 2 h, and shaken every 30 min interval. Then the stomachs were rinsed twice with 0.25 M sucrose solution. The remaining dye complexed with gastric mucus was extracted with 0.5 M magnesium chloride and shaken for 2 h at every 15 min intervals. Diethyl ether was added and centrifuged at 3600 rpm for 10 min. Samples were read at 580 nm using spectrophotometer [19].

### 2.8. Effects of Most Effective Semipurified Extract of MEMM on Antioxidant Enzymes of Gastric Tissues

The gastric tissues were washed thoroughly with ice-cold saline and cut into small pieces. The tissue was then homogenized on ice cold with phosphate-buffered saline (PBS) buffer (50 mM phosphate buffer, pH 7.4) containing a mammalian protease inhibitor cocktail using Teflon Homogenizer. The homogenized gastric tissues were centrifuged at 18 000 ×g for 15 min at 4°C. The supernatant, which was collected and stored at −80°C prior to analysis, was used to measure the activities of catalase (CAT), superoxide dismutase (SOD), glutathione (GSH), prostaglandin E_2_ (PGE_2_), and malondialdehyde (MDA) content. These assays were performed according to the respective manufacturer protocols (Cayman, USA).

#### 2.8.1. Evaluation of Catalase Level

Measurement of CAT level was performed using the catalase assay kit (Cayman Chemical, USA) according to the protocol provided by the manufacturer. Briefly, the collected supernatant was assayed using a microtiter plate wherein each well contains 100 *μ*L of diluted assay buffer, 30 *μ*L of methanol, 20 *μ*L of formaldehyde standard, 20 *μ*L of catalase (positive control), and 20 *μ*L of samples wells, respectively. Approximately 20 *μ*L of diluted hydrogen peroxide was added to all the wells to initiate the reactions for 20 min. The reaction was stopped by adding 30 *μ*L of diluted potassium hydroxide for 10 min at room temperature. Ultimately, 10 *μ*L of catalase potassium periodate was added and incubated for 5 min before the absorbance was read at 540 nm using a plate reader.

#### 2.8.2. Evaluation of Superoxide Dismutase Level

According to the instruction on the SOD kit (Cayman Chemical, USA) provided by the manufacturer, the collected supernatant (10 *μ*L) was mixed with the tetrazolium salt solution (200 *μ*L) to dilute the SOD activity of the supernatants. The reaction was initiated by adding 20 *μ*L of xanthine oxidase and incubated for 20 min in a shaker. The absorbance was then read using the ELISA reader at 440 nm. In this assay bovine erythrocyte SOD (Cu/Zn) was used as the standard. According to this assay procedure, xanthine oxidase and hypoxanthine detected superoxide radicals. In brief, the kit could measure the amount of enzyme that caused 50% dismutation of the superoxide radical.

#### 2.8.3. Evaluation of Glutathione Level

The GSH assay test was assayed according to the protocol provided by the manufacturer (Cayman Chemical, USA). Briefly, the collected supernatant (50 *μ*L) was mixed with a solution containing 0.1 M sodium phosphate, 2 mM ethylenediaminetetraacetic acid (EDTA), 0.4 M 2-(N-morpholino) ethanesulfonic acid, reconstituted NADP+ and glucose-6-phosphate, and reconstituted glutathione reductase and glucose-6-phosphate dehydrogenase. In this study GSH was quantified using glutathione reductase. Following the reduction of hydroperoxides by glutathione peroxidase, oxidized glutathione (GSSG) was produced and used to create the standard curve. The absorbance was read at 405 nm.

#### 2.8.4. Evaluation of Prostaglandin E_2_ Level

The level of PGE_2_ in the collected supernatant was analyzed using the enzyme immunoassay in 96-well plate according to the procedure provided by the manufacturer (Cayman Chemical, USA). Briefly, the supernatant and standards were added to the plate, which was precoated with goat polyclonal anti-mouse immunoglobulin G (IgG). The plate was then incubated with PGE_2_ acetylcholinesterase conjugated with the PGE_2_ Tracer and later applied with Ellman's reagent for 60 min. The kit converted PGE_2_ into Bicyclo PGE_2_ (stable derivative) which was measurable by the kit. The product of this enzymatic reaction had a distinct yellow color and absorbs strongly at 412 nm. Results were calculated using the standard curve which was expressed as picogram per milliliter (pg/mL).

#### 2.8.5. Evaluation of MDA Level

The level of lipid peroxidation in gastric tissues was estimated by determining the MDA content using a thiobarbituric acid reactive substances (TBARS) assay kit (Cayman Chemical, USA). Briefly, 100 *μ*L of SDS solution, 100 *μ*L of the supernatant, and 4 mL color reagent were mixed in a vial and then incubated for 1 h (100°C). This was followed by an incubation of the vial on ice for 10 min after which the vial was centrifuged at 1,600 ×g for 10 min at 4°C. Within 30 min, 150 *μ*L of each vial content was placed on a 96-well plate and the absorbance was read at 530 nm.

### 2.9. Determination of Endogenous Antiulcer Mechanisms of the Most Effective Semipurified Extract of MEMM

#### 2.9.1. Role of Nonprotein Sulfhydryl Groups on the Gastroprotection Exerted by the Most Effective Semipurified Extract of MEMM

To investigate the involvement of the endogenous sulfhydryls compounds in the modulation of gastroprotective effect of the most effective semipurified extract, the procedure by Andreo et al. [20] was used but with slight modifications. Briefly, all groups of rats (*n* = 6) were subjected to intraperitoneal treatment with 10 mg/kg N-ethylmaleimide (NEM). After 30 min, either vehicle (10% DMSO), 100 mg/kg carbenoxolone (CBNX) or 500 mg/kg semipurified extract was orally administered. After 60 min, ethanol (5 mL/kg) was administered orally to all rats for gastric ulcer induction. All groups of rat were euthanized 60 min later and the stomachs were removed to measure the gastric ulcer area.

#### 2.9.2. Role of Nitric Oxide on the Gastroprotection Exerted by the Most Effective Semipurified Extract of MEMM

To investigate the involvement of endogenous NO in the modulation of gastroprotective effect of the most effective semipurified extract of MEMM, the procedure by Andreo et al. [20] was used but with a slight modifications. Six groups of animals were treated intraperitoneally with 70 mg/kg of L-NAME or 500 mg/kg semipurified extract. After 60 min, gastric ulcer in all groups was induced using 5 mL/kg ethanol. All groups of rats were euthanized 60 min later and the stomach was removed for ulcer area measurement.

### 2.10. HPLC Analysis of the Most Effective Semipurified Extract of MEMM and Comparison against the HPLC Profile of Crude Extract MEMM or Pure Flavonoids

The partitions of MEMM, namely, PEMM, EAMM, and AQMM, were also subjected to the HPLC analysis to identify the compound of interest, which could be associated with the extract's gastroprotective effect. In brief, 10 mg of sample was suspended in 1 mL methanol and then filtered through a filter cartridge (pore size of 0.45 *μ*m) prior to use. The filtered sample was then analyzed using the HPLC system (Thermo Scientific Dionex Ultimate 3000 series, Thermo Scientific, Germering, Germany) with Waters 996 photodiode array detector. A Phenomenex RP-Max C_18_ column (4.6 mm i.d. × 250 mm) packed with 5 *μ*m diameter particles was used. The mobile phase used in this procedure contained 0.1% formic acid in water (A) and 0.1% formic acid in acetonitrile (B) and the initial conditions were 90% A and 10% B with a linear gradient reaching 50% B at* t *= 25 min. This condition was maintained for another 5 min and then B was increased from 50% to 95%, within the next 5 min (*t *= 35 min). When the programme reached* t *= 35 min, B was returned to the initial composition (10%) until the programmed reached* t* = 37 min. The flow rate was 1.2 mL/min, injection volume was 10 *μ*L, and the wavelength was 280 nm. The column oven was set at 27°C. Stock solutions of standards references were prepared in methanol at the concentration of 0.3 mg/mL. The chromatography peaks were confirmed by comparing its retention time with those of reference standards and by the respective UV-Vis spectra. All chromatography operations were carried out at ambient temperature and in triplicate.

### 2.11. Statistical Analysis

The data were expressed as means ± SEM and statistical significance was analyzed using ANOVA, followed by Dunnett's post hoc test. Data with the value of *p* < 0.05 were considered statistically significant.

## 3. Result

### 3.1. Pharmacological Activities Observations

#### 3.1.1. Phytochemical Screening of Various Semipurified Extracts of MEMM

The phytochemical screening of the PEMM, EAMM, and AQMM revealed the presence of saponins and tannins, but not alkaloids, in all semipurified extracts (Table 1). Flavonoids were detected only in PEMM and EAMM while triterpenes were detected only in PEMM.

#### 3.1.2. Antioxidant Potential of Various Semipurified Extracts of MEMM

The antioxidant activities of each partition at the concentration of 200 *μ*g/mL are illustrated in Table 2. All partitions exhibited high SOA- and DPPH-radical scavenging activities while only the EAMM followed by AQMM demonstrated high ORAC value. Further analysis showed that EAMM possessed the highest TPC value followed by PEMM and AQMM. According to the standard procedure, a substance with a TPC value that is ≥1000 mg GAE/100 g is considered to have high total phenolic content.

#### 3.1.3. Effects of Various Semipurified Extracts of MEMM on Inflammatory Mediators

The effects of various semipurified extracts of MEMM, at the concentration of 100 mg/mL, on inflammatory mediators, namely, LOX and XO, are shown in Table 3. From the results obtained, all semipurified extracts showed that lack of inhibitory activity towards XO with the highest percentage of inhibition, which is ≤11%, was recorded for EAMM. As for LOX activity, only EAMM and AQMM produced significant percentage of inhibition with the former causing a moderate inhibition with the recorded percentage of inhibition that is greater than 50%.

### 3.2. Gastroprotective Potential of Various Semipurified Extracts of MEMM

#### 3.2.1. Effect of Various Semipurified Extracts of MEMM against Ethanol-Induced Gastric Ulcer

The gastroprotective activity of various semipurified extracts of MEMM against ethanol-induced gastric ulcer in rats is shown in Table 4. All extracts demonstrated significant (*p* < 0.05) reduction in ulcer formation in a dose-dependent manner. However, based on the percentage of ulcer inhibition, EAMM exhibited the greatest protection followed by the AQMM and PEMM with the approximate percentage of inhibition ranging between 15–98%, 7–91%, and 12–67%, respectively. Interestingly, the 250 mg/kg EAMM produced gastroprotection that was equally effective when compared to the 100 mg/kg ranitidine (approximately 60% inhibition).

#### 3.2.2. Macroscopic and Microscopic Findings of Treated Stomachs

**Figure 1:**
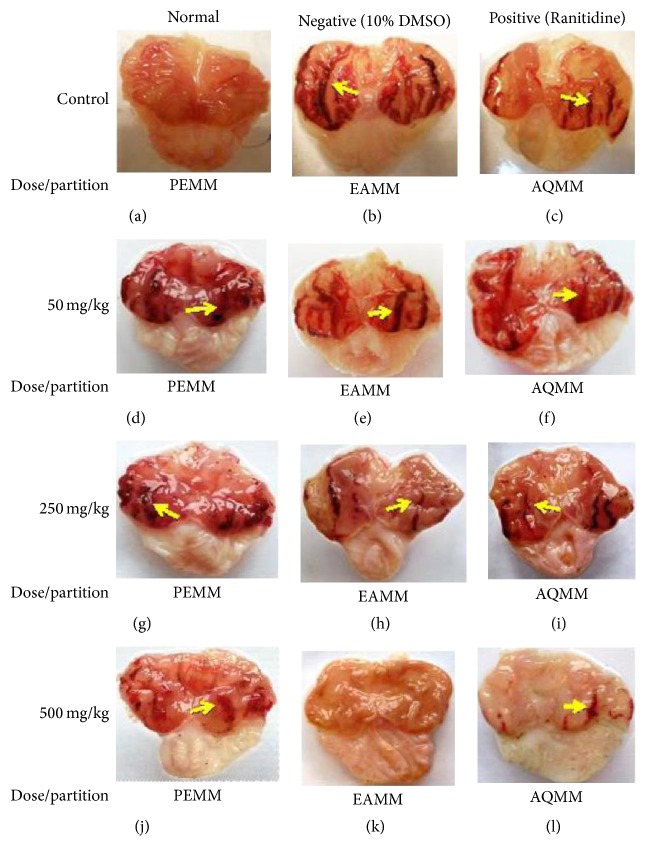
Effect of various partitions, namely, PEMM, EAMM, and AQMM, obtained from methanol extract of* M. malabathricum* (MEMM) against the ethanol-induced gastric ulcer in rats. Arrow (yellow) indicates lesions.

**Figure 2:**
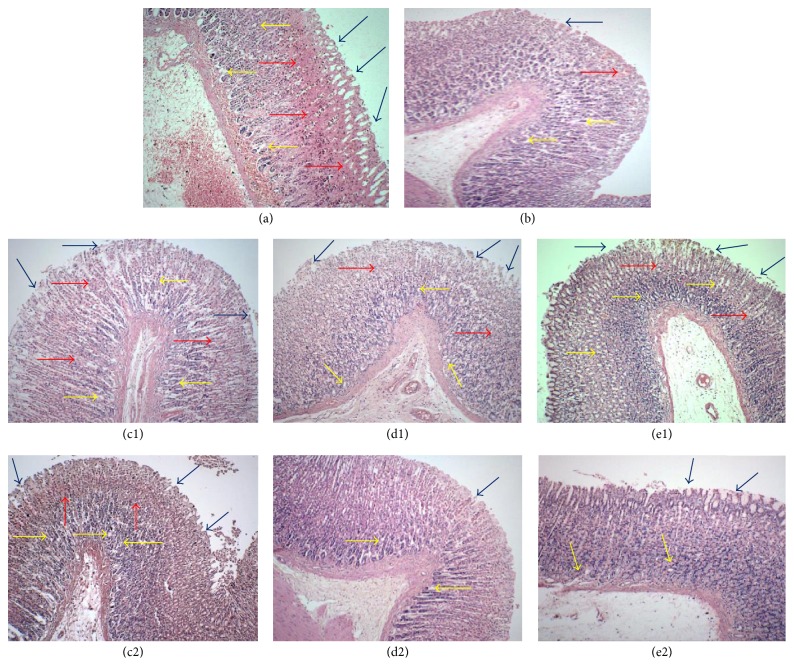
Microscopic analysis of the ethanol-induced gastric ulcer tissues following pretreatment of rats with the respective partition of* M. malabathricum* (magnification at ×20). (a) The negative control group (10% DMSO-pretreated) displayed severe hemorrhage (red arrow), ulcer (blue arrow), and necrosis at mucosa epithelium and edema (yellow arrow) at submucosa layer. (b) The positive control group (ranitidine-pretreated) demonstrated mild ulcer and hemorrhage at mucosa and moderate oedema at submucosa layer. (c1), (d1), and (e1) The groups pretreated with the respective PEMM, EAMM, or AQMM, at the dose of 50 mg/kg, demonstrated severe disruption with ulcer and hemorrhage at mucosa epithelium and severe oedema at submucosa layer with the presence of severe to moderate hemorrhage, ulcer, and necrosis at mucosa epithelium layer. and (c2), (d2), and (e2) At the dose of 500 mg/kg, the group pretreated with EAMM showed the mildest tissue damage when compared to the PEMM or AEMM indicated by the presence of mild ulcer, moderate edema, and the absence of hemorrhage.

Macroscopic examination of the gastric mucosa of the negative control group (ulcer control) showed extensive and visible hemorrhagic necrosis of gastric mucosa (Figure 1(b)) in comparison to the normal untreated group that show no signs of hemorrhage or lesion (Figure 1(b)). Pretreatment with 100 mg/kg ranitidine reduced the formation of hemorrhages and lesions (Figure 1(c)) while pretreatment with PEMM, EAMM, or AQMM at the dose of 50–500 mg/kg also caused a dose-dependent decreased in the severity and visibility of hemorrhagic necrosis of gastric mucosa (Figures 1(d)–1(l)). These findings were further supported by the microscopic observations as shown in Figures 2(a)–2(e2). The negative control group demonstrated severe hemorrhages and necrosis at mucosa epithelium and destruction of the surface epithelium and edema at the submucosa layer (Figure 2(a)). The 100 mg/kg ranitidine-treated group (positive control) demonstrated moderate ulcer formation with the mild hemorrhage seen at the mucosa epithelium and moderate oedema at the submucosa layer (Figure 2(b)). Pretreatment with all semipurified extracts, at the concentration of 50 mg/kg failed to reversed the toxic effect of ethanol as indicated by the presence of severe mucosal disruption with ulcer and hemorrhage seen at the mucosa epithelium and severe oedema at the submucosa level (Figures 2(c1)–2(e1)). Increase in the dose of each partition was found to improve their gastroprotective effect towards the action of ethanol. At 500 mg/kg, pretreatment with EAMM demonstrated almost no disruption at the epithelium mucosa with the presence of mild edema but the absence of hemorrhage (Figure 2(d2)). On the other hand, pretreatment with AQMM exerted mild ulceration at the epithelium mucosa with moderate presence of edema and the absence of hemorrhage (Figure 2(e2)) while pretreatment with PEMM demonstrated moderate disruption of the epithelium mucosa with the presence of moderate hemorrhages and edema (Figure 2(c2)). Overall, the gastric mucosa tissue pretreated by EAMM showed intact appearance of histological structure when compared with the normal control group.

#### 3.2.3. Effect of EAMM against the Pylorus Ligation-Induced Gastric Lesion

Based on the ethanol-induced gastric ulcer test, EAMM was found to exert the most effective gastroprotective activity. This effective semipurified extract was then subjected to further analysis to elucidate the possible mechanisms of gastroprotection. In the first stage of this study, EAMM was tested in the pyloric ligation assay to evaluate its potential in modulating the gastric content parameters such as gastric juice's volume, pH, free and total acidity, and gastric wall mucus content. From the results obtained, EAMM provides gastroprotection by causing significant (*p* < 0.05) reduction in the volume of gastric juice and significant (*p* < 0.05) decrease in the production of free acidity and total acidity. Moreover, EAMM also significantly (*p* < 0.05) increased the pH of gastric juice and enhanced the gastric wall mucus secretion (Table 5).

### 3.3. Effect of EAMM on the Enzymatic and Nonenzymatic Antioxidant Levels of Ethanol-Induced Gastric Ulcer Tissue

Gastric tissue of negative control group treated only with ethanol demonstrated significant (*p* < 0.05) reduction in the level of SOD, CAT, GSH, and PGE_2_ but increase in the level of TBARS, when compared to the normal untreated group. On the other hand, EAMM significantly (*p* < 0.05) reversed the enzymatic and nonenzymatic antioxidant levels of ulcer-bearing gastric tissue when compared to the negative control group (Table 6). As can be seen from the presented table, EAMM increased the level of SOD, CAT, GSH, and PGE_2_ but reduced the level of TBARS when compared to the negative control group.

### 3.4. Role of Endogenous Factors of Gastroprotection on the Action of EAMM

#### 3.4.1. Effect of Nonprotein Sulfhydryl Group on the Gastroprotective Activity of EAMM Assessed Using the Ethanol-Induced Gastric Ulcer in Rats

Investigation on the role of NP-SH in the gastroprotective effect of EAMM was carried out by prechallenging the EAMM or CBNX (positive control) with NEM (NP-SH blocker). From the results obtained, NEM administration was found to significantly (*p* < 0.05) worsen the ethanol-induced gastric ulcer formation in the negative control group. Prechallenging the EAMM- and CBNX-treated group with NEM also resulted in significant (*p* < 0.05) decrease of gastroprotective potential of both compounds (Table 7).

#### 3.4.2. Effect of N-Omega-nitro-L-arginine Methyl Ester on the Gastroprotective Activity of EAMM Assessed Using the Ethanol-Induced Gastric Ulcer in Rats

The role of NO in the modulation of gastroprotective activity of EAMM was also investigated by prechallenging the extract with L-NAME, an NO blocker (Table 7). From the results obtained, the absence of NO following the preadministration of L-NAME significantly (*p* < 0.05) increased the severity of gastric ulcer formed in comparison to the saline-treated negative control group. Further, pretreatment with L-NAME significantly (*p* < 0.05) reversed the gastroprotective action of saline-pretreated EAMM and CBNX.

### 3.5. HPLC Profile of EAMM and Comparison against the HPLC Profile of Crude MEMM and Standard Pure Flavonoids

**Figure 3:**
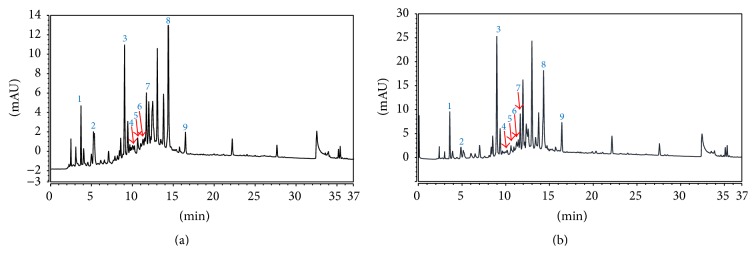
UHPLC analysis of MEMM and EAMM. (a) UHPLC profile of MEMM. (b) UHPLC profile of EAMM.

The HPLC profile of crude methanolic extract (MEMM) and its most effective semipurified partition (EAMM) are shown in Figures 3(a) and 3(b), respectively. The peaks obtained and their respective retention time (*R*_*T*_) in each chromatograms were compared with that of several pure flavonoid-based compounds. From the comparison made, several compounds with their respective *R*_*T*_, namely, gallocatechin (*R*_*T*_ = 3.92 min) (1), epigallocatechin (*R*_*T*_ = 4.91 min) (2), catechin (*R*_*T*_ = 8.89 min) (3), chlorogenic acid (*R*_*T*_ = 10.47 min) (4), caffeic acid (*R*_*T*_ = 10.70 min) (5), quercetin (*R*_*T*_ = 11.63 min) (6), quercetin-3-O-glucoside (*R*_*T*_ = 11.66 min) (7),* p*-coumaric acid (*R*_*T*_ = 14.28 min) (8), and hesperidin (*R*_*T*_ = 16.43 min) (9) were postulated to be presented in MEMM and EAMM.

## 4. Discussion

Ulcer formation on the stomach lining is caused by the imbalance between the protective and aggressive factors and is also associated with living conditions. The major factors of gastric ulcer are bacterial infection by* Helicobacter pylori*, medications such as NSAIDs, chemical factor such as hydrochloric acid or ethanol, and gastric cancer [20]. Meanwhile, minor factors tend to be associated with the lifestyle of the patients such as stress, smoking, spicy food, and nutritional deficiency [20]. Despite the presence of various classes of antiulcer agents, their successes are often associated with various unwanted side effects that limit their usage. Attempts have been made to find an alternative replacement from current medications wherein plant-based compounds have been regarded as an important source of alternative new bioactive compounds.

One of the plants in Malaysia that has been used traditionally by the Malay to treat gastric ulcer is* M. malabathricum* and studies have demonstrated the gastroprotective potential of the aqueous [21], chloroform [22], and methanol [7, 16] extracts of its leaves. The methanolic extract showed the ability to attenuate ethanol- and indomethacin-induced gastric ulcer in rats [16]. Moreover, the extract showed a significant reduction in the volume and acidity of the gastric juice while increasing the pH and gastric mucus wall content by using the pylorus ligation model in rats [7].

The MEMM also modulates the level of several enzymatic and nonenzymatic antioxidant system within the gastric tissue wherein the levels of SOD, glutathione peroxidase (GTP), and glutathione reductase (GTR) were increased but the levels of CAT, myeloperoxidase (MPO), and TBARS were decreased. Moreover, the gastroprotective activity of MEMM was reduced by an inhibitor of NO synthase and SH blocker. Methanol is an intermediate solvent, which is able to extract all the polar-, nonpolar-, and intermediate-based biocompounds that exert the gastroprotective activity. This has triggered our interest to segregate the biocompounds according to their polarity to further understand their activity.

The PEMM, EAMM, and AQMM were tested for their antioxidant, anti-inflammatory, and gastroprotective potentials using various standard assays. Although all partitions exerted remarkable antioxidant activity when assessed using the SOA- and DPPH-radical scavenging assays, EAMM exerts the most effective activity when measured using the ORAC assay and possesses the highest TPC value with significant LOX- but not XO-mediated in vitro anti-inflammatory action. All partitions were tested using the ethanol-induced gastric ulcer model in rats and macroscopic observations demonstrated that EAMM exert the most effective gastroprotective activity, followed by AQMM and PEMM. The EAMM exerted almost complete protection (≈98% reduction in ulcer area formation) against the action of ethanol and was further supported by the microscopic observations, which showed only slight epithelial sloughing off but no presence of hemorrhage, edema, or necrosis. Taking these findings into consideration, the EAMM was determined as the most effective partition and subjected to further analysis. In the pylorus ligation assay, EAMM was shown to modulate several parameters of gastric content wherein the semipurified extract reduces the volume and free acidity and total acidity while increasing the pH of gastric juice. Moreover, EAMM was also found to enhance the secretion of gastric wall mucus. These findings suggest that EAMM triggered gastroprotective effect partly by reducing the volume and acidity and at the same time increasing the pH of the gastric juice. Further attempt to elucidate the ability of EAMM to affect the enzymatic and nonenzymatic antioxidant system within the gastric tissues following ethanol-induced ulcer formation revealed the ability of the semipurified extract to modulate the enzymatic and nonenzymatic antioxidant system. EAMM was found to increase the level of SOD, CAT, GSH, and PGE_2_ while reducing the level of TBARS. It also revealed the role of endogenous factors such as NP-SH group and NO in the modulation of gastroprotective activity of EAMM. The NEM (NP-SH blocker) decreases the extract's gastroprotective activity remarkably and the ability of L-NAME (NO blocker) to reverse the EAMM gastroprotection suggests the role of NO in the EAMM's mechanisms of gastroprotection.

The pathogenesis of gastric ulcers have been widely known to involve oxidative stress. Ingestion of these necrotic agents may trigger the formation and release of free radicals or reactive oxygen species (ROS), which are known to contribute to the damage of gastric mucosa. Therefore, any compounds that were able to reduce oxidative stress or possess antioxidant potential are essential to the gastrointestinal tract as they can provide protection against the action of necrotic agents such as ethanol [23]. Those antioxidant biocompounds may act as radical scavengers thus protecting the gastric mucosa from oxidative damage. EAMM exert the most notable antioxidant potential and possess the highest TPC value in comparison to other partitions, which makes it a potential candidate to further investigate the antiulcer activity of* M. malabathricum*. The antioxidant and free radical scavenging properties of EAMM may have contributed to the observed gastroprotective effect.

The role of anti-inflammatory action in the modulation of EAMM induced gastroprotection should also be considered based on the fact that* M. malabathricum* exerts an effective anti-inflammatory activity when assessed using various COX-mediated animal inflammatory models [24, 25]. The EAMM was found to cause over 50% inhibition of LOX-mediated in vitro inflammation, therefore, suggesting the anti-inflammatory potential against the LOX-mediated inflammation that might contribute towards the effective gastroprotection of EAMM [26]. LOX is known to take part in the oxidation of arachidonic acid into a family of eicosanoid inflammatory mediators known as leukotrienes. Leukotrienes are believed to contribute to gastric mucosal damage by promoting tissue ischaemia and inflammation and play an imperative role in blood coagulation and gastrointestinal tract irritation [27]. Therefore, inhibition of LOX as seen with EAMM is believed to be vital in attenuating the formation of ethanol-induced gastric ulcer [28].

Despite the ability to exert anti-inflammatory activity, EAMM has been shown in the presence study to exert antiulcer activity and was effective in increasing the level of PGE_2_ following subjection to the ethanol-induced gastric ulcer assay. The discrepancy in the action of EAMM is therefore worth discussing. Prostaglandins synthesis depends on the activity of COXs, which exist as distinct isoforms referred to as COX-1 and COX-2. COX-1, which is the dominant source of prostanoids that subserve housekeeping functions, such as gastric epithelial cytoprotection and homeostasis, expressed constitutively in most cells. On the other hand, COX-2 which is the more important source of prostanoid formation in inflammation and in proliferative diseases is induced by inflammatory stimuli, hormones, and growth factors. There is a dramatic increase of COX-2 expression upon provocation of inflammatory cells and in inflamed tissues. However, both enzymes contribute to the generation of autoregulatory and homeostatic prostanoids and both can contribute to prostanoid release during inflammation. Thus, with regard to its anti-inflammatory activity, EAMM is suggested to act preferentially towards inhibiting the COX-2 action, which lead to the inhibition of PGE_2_ synthesis and inflammatory action. In terms of its antiulcer activity, EAMM is postulated to activate the COX-1 action leading to increase synthesis of local PGE_2_ and PGI_2_, which provides protection towards the gastroduodenal epithelial integrity. The disruption of this pathway via inhibition of COX-1 will lead to ulcer formation. Moreover, EAMM may be able to activate the gastroduodenal epithelial COX-2-dependent prostanoids (i.e., PGE_2_ and PGI_2_) that hasten ulcer healing. Both PGs, in particular, are vasodilators in the gastrointestinal mucosa, which may increase mucus production and reduce acid and pepsin levels in the stomach, thereby contributing to the gastric mucosal defense and facilitate the repair of preexisting ulcers in the gastrointestinal mucosa [29, 30].

The ethanol-induced gastric ulcer model is frequently used to evaluate the antiulcerogenic activity of drugs. Ethanol acts by rapidly penetrating the gastric mucosa and damaging the cell and plasma membranes leading to increase in intracellular membrane permeability to sodium and water. This, in turn, leads to massive accumulation of calcium that clarifies the pathogenesis of gastric mucosal injury [31]. Continuous ingestion of ethanol resulted in the development of gastric lesions in the form of multiple hemorrhagic red bands of different sizes along the glandular stomach. The gastric mucosal lesions incited by ethanol ingestion might decelerate the mechanism of gastric defense [32], which include the depletion of gastric mucus content, mucosal cell injury, and damaged mucosal blood flow [33]. Other than direct action on the gastric mucosa, ethanol also augments the production of ROS (i.e., superoxide anion and hydroxyl radicals) and enhances release of arachidonate metabolites [34, 35]. The ROS, in particular triggers lipid peroxidation that will cause disturbance in cellular activities leading ultimately to membrane cell damage, cell death, exfoliation, and epithelial erosion. This may explain the ability of EAMM to attenuate the ethanol-induced gastric ulcer formation due to its high antioxidant and anti-inflammatory capacities.

The ability of any compound to control the contraction and relaxation of gastric circular muscles might also contribute to the enhancement of gastroprotective effect [36, 37]. The contraction of circular muscles of the rat fundus strip caused by ethanol ingestion may lead to compression of the mucosa at the crest of the mucosal folds, causing ulceration and necrosis [36]. On the other hand, circular muscles relaxation will cause the gastric mucosa folds to flatten as part of the gastric mucosa defense by increasing the exposure of mucosal area to necrotizing agents while reducing the volume of gastric irritants on the rugal crest [37]. From the macroscopic observation, a flattening of the mucosal folds was observed following pretreatment with EAMM. This suggest that the EAMM also promotes gastroprotective action by decreasing the gastric motility as these alterations may also contribute to the development and prevention of gastric lesions.

The possible mechanisms of gastroprotection involving the role of NO and NP-SH were also investigated using the ethanol-induced gastric ulcer assay in rats. The rats were pretreated with L-NAME or NEM followed by EAMM before inducing with ethanol, respectively. Both the L-NAME and NEM caused significant reduction in the gastroprotective activity of EAMM suggesting that the semipurified extract works in the presence of NO and NP-SH. NO is one of the vital defensive endogenous factors in the gastric mucosa [38] and is important for the modulation of gastric mucosal integrity. Moreover, NO is vital for the regulation of mucus secretion, acid and alkaline secretion, and gastric mucosal blood flow [39]. The endothelium is the main and most preferred target of gastric ethanol damage [40]. In addition, L-NAME when given systemically inhibits NO synthesis/action leading to an increase in systemic blood pressure and the vasoconstriction of several vascular beds that damage the gastric mucosa and its endothelium [41]. Pretreatment of rats with L-NAME followed by EAMM reversed the gastroprotective effects exerted by the semipurified extract against ethanol-induced injury. These findings indicate the possible participation of the NO-mediated system in the gastroprotective activity elicited by EAMM. Mucus is important in gastroprotection as it helps to strengthen the mucosal barrier against harmful agents. At the molecular level, the mucus subunits are connected via disulfide bridges and reduction in this bridges will cause the mucus to be more water soluble [42]. The disulfide bridges help to maintain continuous adherence of the stable, undisturbed mucus layer, which serves to protect the underlying mucosa from proteolytic digestion [43]. The NP-SH compound exerts its protective effects against prooxidant agents by binding the free radicals synthesized following the ingestion of noxious agents [40].

In regard to the phytochemical constituents of EAMM, several classes of biocompounds were detected, namely, saponins, tannins, flavonoids, and steroids. This finding was concurrent with our previous report on the presence of those classes of biocompounds in the crude MEMM [44]. A number of biocompounds from the classes of flavonoids, saponins, and tannins are known to act as antioxidants [45–47] and anti-inflammatory [48–50] and are, therefore, suggested to act synergistically to exert both activities in all models used. The synergistic action of those biocompounds is believed to be accountable towards the effectiveness of gastroprotective activity shown by EAMM. Therefore, the presence of these phytochemical substances may partly protect against gastric lesions by enhancing the antioxidant and anti-inflammatory activities of EAMM. In addition, flavonoid-based biocompounds have been reported to promote gastroprotection by increasing the gastric blood flow, stimulating the synthesis of mucosubstances of the gastric mucosa, increasing the PGs content and mucus thickness [51, 52]. Flavonoid-based biocompounds were also reported to promote formation of the gastric mucosa, inhibit pepsinogen production, decrease acid mucosal secretion, and reduce ulcerogenic lesions [53]. The ability of tannin-based biocompounds to prevent ulcer formation is by promoting protein precipitation layer on the ulcer site to form a protective pellicle, which helps to prevent the absorption of toxic substances and withstand the effects of proteolytic enzymes [54–56]. Meanwhile, the saponin-based biocompounds have been reported to demonstrate its gastroprotective effect by activating the mucus membrane protective factors [57] and by selectively inhibiting PGF_2*α*_ [31].

Several types of bioactive compounds have been identified in the MEMM as well as EAMM [6]. The closest findings that mimic the present extract, MEMM, were made by Nazlina et al. [58], who successfully isolated rutin, quercitrin, and quercetin using the TLC assay while those that mimic the present partition, EAMM, were made by Susanti et al. [59], who managed to isolate quercetin and quercitrin. Recent study on the hepatoprotective activity of MEMM also demonstrated the presence of rutin and quercitrin. Interestingly, quercetin, quercitrin, and rutin have been reported to play a role in gastroprotection elsewhere [53, 60, 61]. Therefore, these compounds are suggested to act together synergistically to produce the observed gastroprotective effect. Following the latest HPLC analysis of MEMM and EAMM, which used different set of HPLC equipment, conditions, eluants, and so forth in comparison to our previous study on MEMM [7, 44], several bioactive flavonoid-based compounds were identified, namely, gallocatechin (1), epigallocatechin (2), catechin (3), chlorogenic acid (4), caffeic acid (5), quercetin (6), quercetin-3-*O*-glucoside (7),* p*-coumaric acid (8), and hesperidin (9). Interestingly, some of these compounds like catechin [62], chlorogenic acid [63], caffeic acid [64], quercetin [65], and hesperidin [66] have been reported to exert antiulcer activity and are, therefore, expected to act synergistically to demonstrate the antiulcer activity as observed in MEMM and EAMM.

## 5. Conclusion

In conclusion, the ethyl acetate partition of MEMM or EAMM demonstrates the most effective gastroprotective activity against ethanol-induced gastric ulcer model, which could be attributed to the extract's (i) high antioxidant and anti-inflammatory activities; (ii) capability to modulate the gastric tissue's enzymatic and nonenzymatic antioxidant system; (iii) potential to regulate the PGE_2_ synthesis, and (iv) ability to work via pathways involving the NO and NP-SH. Moreover, this activity could be plausibly linked to the presence of gastroprotective agents such as catechin, chlorogenic acid, caffeic acid, quercetin, and hesperidin, which might act synergistically to produce the observed activity.

## Figures and Tables

**Table 1:** Phytochemical screening of PEMM, EAMM, and AQMM.

Phytoconstituents	PEMM	EAMM	AQMM
Alkaloids	—	—	—
Saponins	1+	2+	2+
Flavonoids	1+	1+	—
Tannins	1+	2+	3+
Triterpenes	2+	—	—
Steroids	3+	1+	—

Phytoconstituent content scoring.

Alkaloids: + negligible amount of precipitate; ++ weak precipitate; +++ strong precipitate.

Saponins: + 1-2 cm froth; ++ 2-3 cm froth; +++ >3 cm froth.

Flavonoids, tannins, and triterpenes: + weak color; ++ mild color; +++ strong color.

**Table 2:** Antioxidant activity and TPC value of various partitions of MEMM measured using different assay.

Sample	SOA scavenging (%)	DPPH radical scavenging (%)	ORAC Value (*μ*M TE/100 g)	TPC (mg/100 g GAE)
Sample concentration	200 *μ*g/mL	200 *μ*g/mL	200 *μ*g/mL	200 *μ*g/mL
Standard	Superoxide dismutase 6 × 10^−3^ U/mL	Ascorbic acid (AA) 200 *μ*g/mL	Trolox standard curve	Gallic acid (GAE) standard curve
PEMM	100 ± 0.0 (H)	96.29 ± 1.9 (H)	32,000 ± 3,000	279.53 ± 7.93
EAMM	98.03 ± 0.74 (H)	97.94 ± 0.4 (H)	198,000 ± 9,800	963.10 ± 35.96
AQMM	98.17 ± 1.83 (H)	98.95 ± 0.1 (H)	185,000 ± 7,300	177.57 ± 14.26

Data expressed as mean ± SEM.

SOA scavenging and DPPH radical scavenging; H: high (70–100%), M: moderate (50–69%), and L: low (0–49%).

TPC value > 1000 mg GAE/100 g is considered higher total phenolic content.

The ORAC value of triplicate wells in duplicate experiments, SEM < 20%.

**Table 3:** Anti-inflammatory effect of various partitions of MEMM against in vitro xanthine oxidase and lipoxygenase assays.

Sample concentration 100 mg/mL	Xanthine oxidase assay	Lipoxygenase assay
	(%)	(%)
PEMM	3.32 ± 1.68 (L)	NA
EAMM	10.23 ± 2.58 (L)	59.15 ± 4.43 (M)
AQMM	NA	32.84 ± 3.65 (L)

Data expressed as mean ± SEM.

Note: H: high (71–100%), M: moderate (41–70%), L: low (0–40%), and NA: not active.

**Table 4:** Gastroprotective effect of various partitions of MEMM against ethanol-induced in rats.

Pretreatment	Dose (mg/kg)	Ulcer area (mm^2^)	Gastroprotection (%)
10% DMSO	—	27.00 ± 0.71	—

Ranitidine	100 mg	9.80 ± 1.11^*∗*^	63.70

PEMM	50 mg	23.60 ± 1.36	12.59
	250 mg	18.00 ± 0.71^*∗*^	33.33
	500 mg	12.40 ± 0.68^*∗*^	54.07

EAMM	50 mg	19.80 ± 1.07	26.67
	250 mg	10.00 ± 1.18^*∗*^	62.96
	500 mg	0.80 ± 0.20^*∗*^	97.04

AQMM	50 mg	22.80 ± 0.86	15.56
	250 mg	15.40 ± 0.68^*∗*^	42.96
	500 mg	3.80 ± 0.37^*∗*^	85.93

Data were expressed as mean ± SEM and analyzed by one-way ANOVA followed by Dunnett's post hoc test (*n* = 6).

^*∗*^ *p* < 0.05 as compared to control (10% DMSO).

**Table 5:** Gastroprotective effect of EAMM against the pylorus ligation assay.

Pretreatment	Dose (mg/kg)	Gastric juice (mL)	pH	Free Acidity (mEq/L)	Total acidity (mEq/L)	Gastric wall mucus (Alcian blue *μ*g/g wet tissue)
10% DMSO	—	9.67 ± 0.67	1.51 ± 0.12	1061.00 ± 205.70	1513 ± 122.30	263.10 ± 35.43

Ranitidine	100	2.30 ± 0.37^*∗*^	4.18 ± 0.81^*∗*^	243.00 ± 39.56^*∗*^	644.00 ± 89.61^*∗*^	592.60 ± 31.84^*∗*^

EAMM	50	13.33 ± 1.15	1.99 ± 0.17	528.00 ± 37.70^*∗*^	692.60 ± 95.98^*∗*^	404.10 ± 48.31
	250	6.75 ± 0.93	3.40 ± 0.49^*∗*^	319.20 ± 46.76^*∗*^	553.40 ± 43.89^*∗*^	636.90 ± 41.33^*∗*^
	500	5.17 ± 0.50	4.00 ± 0.27^*∗*^	292.10 ± 47.87^*∗*^	579.50 ± 38.42^*∗*^	659.30 ± 41.15^*∗*^

Data were expressed as mean ± SEM and analyzed by one-way ANOVA followed by Dunnett's post hoc test (*n* = 6).

^*∗*^ *p* < 0.05 as compared to control (10% DMSO).

**Table 6:** Effect of EAMM on SOD, CAT, GSH, PGE_2_, and TBARS level in rat gastric tissues.

Pretreatment	Dose	SOD	Catalase	GSH	TBARS	PGE_2_
	(mg/kg)	(U/mg protein)	(nmol/min/mL)	(*μ*M/mg protein)	(*μ*mol/mL)	(ng/mg)
Normal (untreated)	—	2.51 ± 0.07^*∗*^	127.60 ± 0.62^*∗*^	13.57 ± 0.55^*∗*^	0.12 ± 0.00^*∗*^	11.30 ± 0.67^*∗*^

10% DMSO	—	1.28 ± 0.04	198.21 ± 1.60	2.51 ± 0.34	0.37 ± 0.03	4.74 ± 0.25

Ranitidine	100	1.79 ± 0.04^*∗*^	117.80 ± 0.80^*∗*^	4.69 ± 0.40^*∗*^	0.16 ± 0.09^*∗*^	8.27 ± 1.01^*∗*^

EAMM	50	1.30 ± 0.07	104.40 ± 1.48^*∗*^	3.29 ± 0.36	0.22 ± 0.01	4.75 ± 0.31
	250	1.71 ± 0.10^*∗*^	122.30 ± 1.62^*∗*^	5.12 ± 0.29^*∗*^	0.16 ± 0.08^*∗*^	8.37 ± 0.84^*∗*^
	500	1.71 ± 0.12^*∗*^	120.30 ± 1.91^*∗*^	5.93 ± 0.42^*∗*^	0.16 ± 0.01^*∗*^	5.94 ± 0.61^*∗*^

Data were expressed as mean ± SEM and analyzed by one-way ANOVA followed by Dunnett's post hoc test (*n* = 6).

^*∗*^ *p* < 0.05 as compared to control (10% DMSO).

**Table 7:** Gastroprotective effect of EAMM following pretreatment with L-NAME or NEM assessed using the ethanol-induced gastric ulcer model.

Pretreatment	Treatment	Dose (mg/kg)	Ulcer area (mm^2^)
Saline	10% DMSO	—	27.68 ± 1.69
	CBXN	100	6.83 ± 0.60^a^
	EAMM	500	1.67 ± 0.33^a^

L-NAME	10% DMSO	—	36.67 ± 2.78^a^
	CBXN	100	12.00 ± 0.73^bc^
	EAMM	500	8.83 ± 0.60^bd^

NEM	10% DMSO	—	41.67 ± 1.75^a^
	CBXN	100	18.50 ± 2.26^ef^
	EAMM	500	11.25 ± 0.54^eg^

Data were expressed as mean ± SEM and analyzed by one-way ANOVA followed by Dunnett's post hoc test (*n* = 6).

^a^ *p* < 0.05 as compared to control (saline + 10% DMSO).

^b^ *p* < 0.01 as compared to control (L-NAME + 10% DMSO).

^cf^ *p* < 0.05 as compared to control (saline + CBXN).

^dg^ *p* < 0.05 as compared to control (saline + EAMM).

^e^ *p* < 0.05 as compared to control (NEM + 10% DMSO).
